# Two-step deposition of Al-doped ZnO on p-GaN to form ohmic contacts

**DOI:** 10.1186/s11671-017-2239-x

**Published:** 2017-07-26

**Authors:** Xi Su, Guozhen Zhang, Xiao Wang, Chao Chen, Hao Wu, Chang Liu

**Affiliations:** Key Laboratory of Artificial Micro- and Nano-structures of Ministry of Education, School of Physics and Technology, Wuhan University, Wuhan, 430072 People’s Republic of China

**Keywords:** AZO, PAD, ALD, Ohmic contacts

## Abstract

Al-doped ZnO (AZO) thin films were deposited directly on p-GaN substrates by using a two-step deposition consisting of polymer assisted deposition (PAD) and atomic layer deposition (ALD) methods. Ohmic contacts of the AZO on p-GaN have been formed. The lowest sheet resistance of the two-step prepared AZO films reached to 145 Ω/sq, and the specific contact resistance reduced to 1.47 × 10^−2^ Ω·cm^2^. Transmittance of the AZO films remained above 80% in the visible region. The combination of PAD and ALD technique can be used to prepare p-type ohmic contacts for optoelectronics.

## Background

Nowadays GaN-based compound semiconductors have already achieved substantial progresses and have been comprehensively utilized in high temperature, high power, and high-frequency devices [1, 2], in which ohmic contacts are crucial for good device performance. So far, it is still very difficult to realize ohmic contacts to p-type GaN [3, 4]. Over a long period of time, oxidized Ni/Au [5], Ni/Pd/Au [6] and Pd/Ni [7] etc. are common solutions, although Au contacts are opaque, expensive and unstable at high temperature. Therefore, seeking an alternate that is thermally stable and transparent is imminent. Up till now, transparent conductive oxides (TCO) such as Al-doped ZnO (AZO) and Sn-doped In_2_O_3_ (ITO) have been widely used as the electrode materials. However, both tin and indium are costly and unfriendly to the environment. In contrast, AZO is promising due to its high-transparency, low-resistance, low-cost and non-toxicity [8–10]. It has been reported that AZO films can be prepared by many methods such as atomic layer deposition [8], sputtering [11], e-beam evaporation [12], pulsed laser deposition [13] and sol-gel [14]. Due to the difference of the electron affinities between AZO (4.7 eV) and p-GaN (7.5 eV) [15], it is difficult to achieve ohmic contacts by directly depositing AZO onto GaN [16], although it was reported that after annealing the deposited AZO films on p-GaN resulted in ohmic behavior [17, 18]. To solve the problem, several kinds of interlayers have been introduced, e.g., NiO [16], Ag nanoparticles [19, 20], p-InGaN [21], Pt layer [22] and InON nanodots [23].

In this work, a two-step method was developed to achieve ohmic contacts between AZO and p-GaN. The first step is to grow AZO thin films as the interlayer by polymer assisted deposition (PAD). AZO films with different metal cation mole ratios of aluminum to zinc (n_Al_ : n_Zn_) were directly grown on p-GaN. The influence of different growing temperatures and annealing temperatures on the crystalline quality and the conductivity of the films were extensively studied. The second step is to grow AZO thin films by atomic layer deposition (ALD) on the top of the PAD-grown AZO. The AZO films show a favorable (002) orientation with good crystalline quality, a good ohmic behavior on p-GaN and high transmittance. PAD-AZO layer ensured ohmic contact while ALD-AZO layer decreased the specific contact resistance and the sheet resistance to make it usable.

## Methods

PAD is a new chemical-solution deposition method developed in recent years and has been proven to be a practical method to grow metal oxide films with good crystalline quality on large scale of regular and irregular surfaces with very low cost [24–27]. PAD-AZO films (about 30 nm) were grown directly on p-GaN following the standard procedures of PAD method [24]. The solution of the PAD-AZO films was prepared by blending two separate solutions of Zn and Al bound to polymers. The concentrations of Zn (3.06 × 10^−4^ mol/mL) and Al (7.41 × 10^−5^ mol/mL) in these two solutions were characterized by inductively coupled plasma-atomic emission spectrometer (ICP-AES), and the different volumes of the two solutions were mixed together, forming AZO precursors with different mole ratios of Al to Zn. The mixed solution was spin coated onto substrates at 3000 rpm for 40 s, and then preheated at 60 °C in air for 10 min on a hot plate. The films were then heated at 500, 600, 700 and 800 °C for 2 h in air. ALD method was used as the second step to increase the conductivity. The ALD-AZO films (about 120 nm) were deposited at 150 °C by using Beneq TFS-200, and the details of the ALD process can be found in our previous work [8–10]. The substrates in this experiment were p-GaN (the carrier concentration was about 1.2 × 10^17^ cm^−3^) and quartz glass. Surface topography was measured by atomic force microscopy (AFM, Bruker Multimode 8). Crystallinity and orientation of these films were measured by x-ray diffraction (XRD, Bede D1). Transmission of the films was measured by ultraviolet-visible spectrophotometer (UV-2550; Shimadzu, Kyoto, Japan). Electrical resistivity was measured by a hall measurement (Model 7707A, Lake Shore, USA) using a van der pauw geometry. The AZO films were etched by phosphoric acid for about 2 min (the etching speed was about 100 nm/min) with a 1 cm^2^ mask on them to form the square shape. After etching, four lead wires were connected onto the four square electrodes. Specific contact resistance and current-voltage (I-V) curves were measured by using circular transmission line model (CTLM) method. CTLM patterns were defined on substrate by using a standard photolithographic technique before grown.

## Results and discussion

**Fig. 1:**
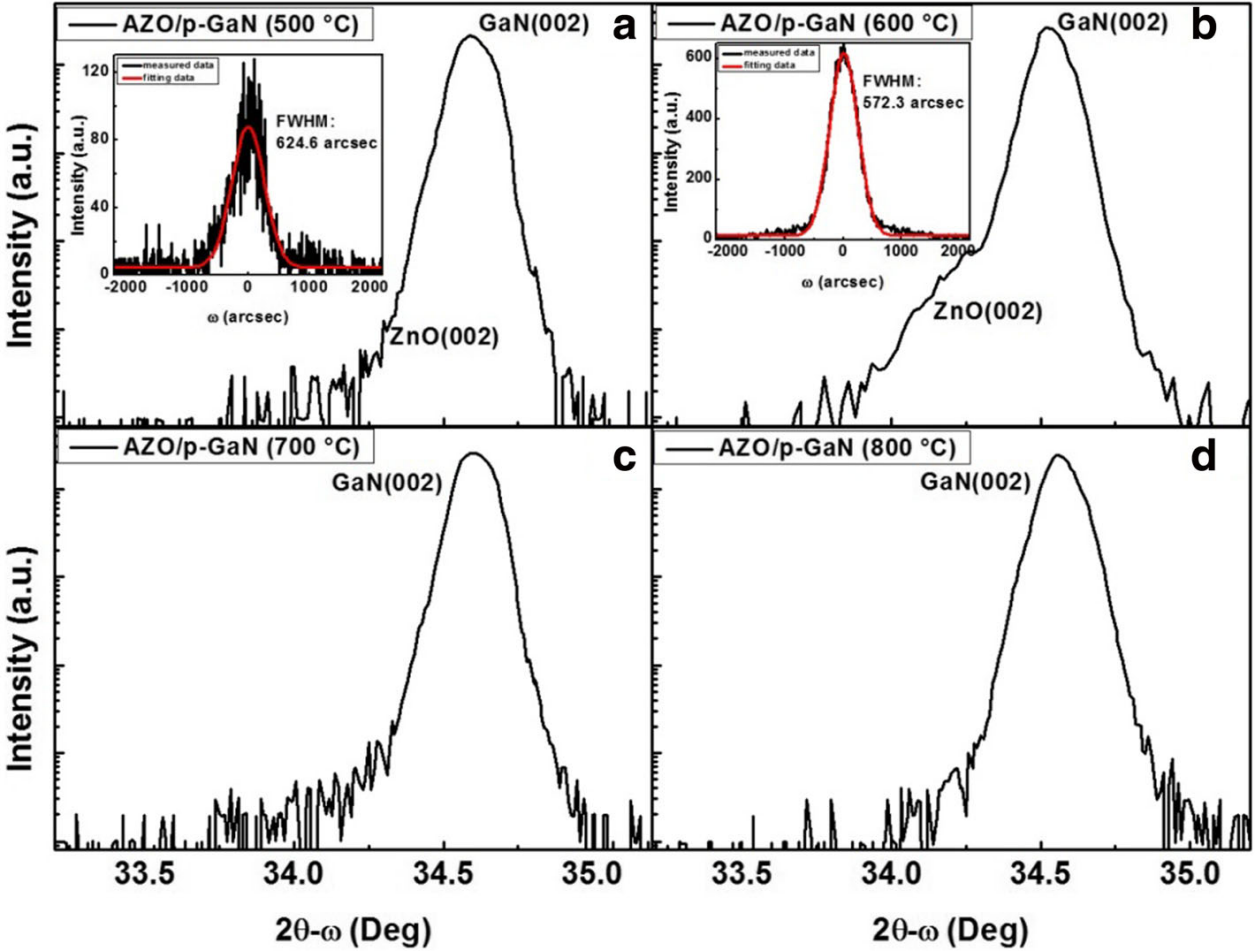
X-ray diffraction spectra of AZO films deposited directly on p-GaN substrate by PAD method in different temperature. **a** 500 °C; **b** 600 °C; **c** 700 °C and **d** 800 °C. The inner graphs of (**a**) and (**b**) show the rocking curve of the 002 diffraction peak of AZO

Figure 1 shows the x-ray diffraction (XRD) spectra of the AZO films deposited directly on p-GaN substrates by the PAD method. The grown temperatures were set at 500 (Fig. 1a), 600 (Fig. 1b), 700 (Fig. 1c) and 800 °C (Fig. 1d), respectively, and the compositions of all samples were kept the same (n_Al_:n_Zn_ = 9:100) It can be observed from Fig. 1b that the main peak was indexed to GaN (002), while the shoulder was attributed to AZO (002). The AZO films grow by PAD method show a favorable c-axis orientation. The AZO films grown at 500 °C and 600 °C show good crystallinity, and the full width at half maximum (FWHM) of the (002) rocking curve were 625 and 572 arcsec. Obviously, the growing temperature plays a crucial role for the growth of AZO. At 500 °C, the polymer just burned out and might impact on crystallization. When the temperatures were 700 °C and 800 °C, decomposition of AZO happened and this was the reason of disappearance of the shoulder peak. It can be explained that the good crystalline quality of AZO is attributed to two factors: The first is related to the lattice match between ZnO and GaN, their mismatch is less than 2% according to the following formula: |a_e_ − a_s_|/a_e_, where a_s_ stands for the lattice constant of the substrate of GaN, a_e_ stands for the lattice constant of the epilayer of ZnO. The second is owing to the optimized growth temperature of 600 °C at which the polymer was decomposited and ZnO crystallized along c-axis.

**Fig. 2:**
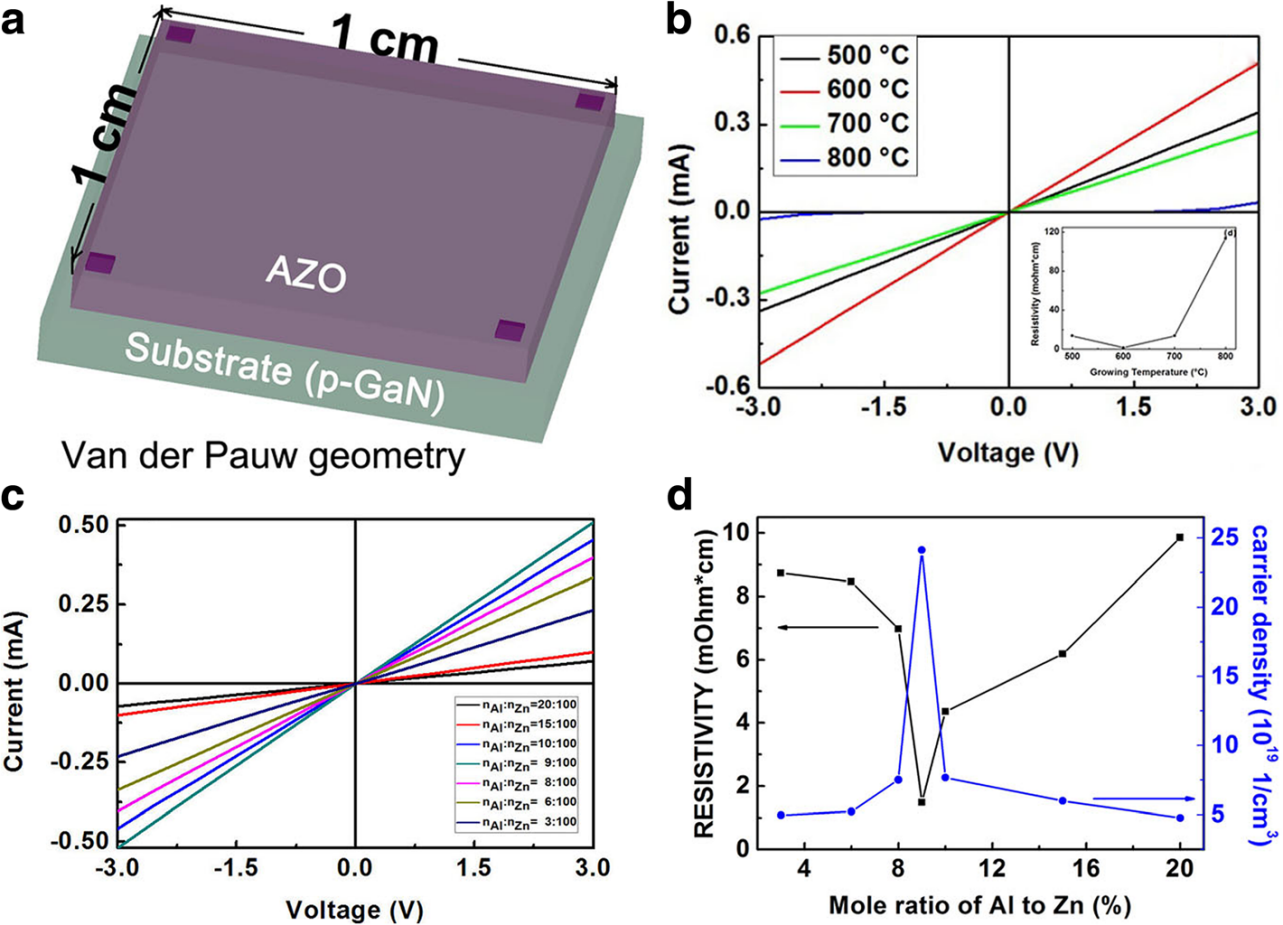
**a** The sketch graphs demonstrating van der pauw geometry. **b** The resistivity of AZO films with different growth temperatures (500, 600, 700 and 800 °C). The inner graph of (**b**) shows the temperature dependence of the resistivity. **c** Current-voltage characteristics of different mole ratios of Al to Zn. **d** The resistivity and carrier density vs different mole ratios of Al to Zn

Figure 2a show schematic diagrams of the structures for van der pauw geometry. In order to acquire more reasonable results, before all electrical tests, indium electrodes were spot welded on AZO surface. The AZO was n-type semiconductor and ohmic contact between AZO and indium electrode was easy to achieve. Figure 2b and the inner graph of 2 (b) shows I-V characteristics and the resistivity of the AZO films that grown at different temperatures (500, 600, 700 and 800 °C). When the growth temperatures of the PAD-AZO were set at 500, 600 and 700 °C, the contacts between PAD-AZO and p-GaN were ohmic. When the growth temperature was at 600 °C, the sheet resistance decreased with the increase of the growth temperature, when the growth temperature was about 600 °C, the sheet resistance reached the lowest value (740 Ω/sq), and it increased with the increase of the growth temperature. Basically, the resistivity of the electrode needs to be as low as possible. The Fig. 2c shows the I-V characteristics of AZO films with different mole ratios of Al to Zn. It can be observed that all samples exhibited linear I-V characteristics, which implied that the contacts of the as-deposited AZO on p-GaN were ohmic. Figure 2d shows the resistivity and carrier density of the AZO films versus different mole ratios of Al to Zn. The lowest sheet resistance of the PAD-AZO was about 740 Ω/sq. It revealed that when the mole ratio of Al to Zn was below 9%, the resistance decreased with the increase of the mole ratio of Al to Zn, and when the mole ratio of Al to Zn was over 9%, the resistance then increased with the increase of the mole ratio of Al to Zn. And the variation tendency of the AZO films was similar to Fig. 2c. Obviously, self-compensation occurred at high doping range. It is obviously that the conductivity remains to be improved. It can be known from the equation R_sh_ = ρ/t (where ρ stands for resistivity and t stands for thickness of the film) that the sheet resistance (R_sh_) decreases with the increase of the thickness of the film, hence the thickness of the PAD-AZO should be increased to reduce the resistivity. Due to the characteristics of the PAD method, in order to improve the thickness of AZO films, multiple spin coating and heat treatment were inevitable [28]. However, after several times of heat treatment, it was found that the resistance increased, the sheet resistance reached 7600 Ω/sq. when the thickness of PAD-AZO was about 150 nm. The increase of the resistance may be caused by multiple heat treatments, so other solutions need to be found. Our group’s previous work indicated the resistivity of ALD-AZO films can be relatively low [8–10], so ALD method was added in.

**Fig. 3:**
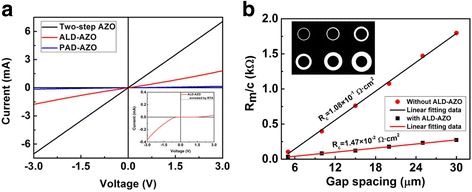
**a** Current-voltage characteristics of PAD-AZO, ALD-AZO and two-step AZO grown on p-GaN. The inner graph of (**a**) shows the I-V curve of ALD-AZO that annealed by RTA in N_2_ for 60 s. **b** shows the raw data and linear fitting data of the specific contact of the PAD-AZO (without ALD-AZO) and two-step AZO (with ALD-AZO) to extract specific contact resistance, the inner graph of (**b**) shows the structure of CTLM

Figure 3a shows I-V characteristics of the PAD-AZO, the ALD-AZO and two-step AZO deposited on p-GaN, the inner graph shows I-V characteristics of the ALD-AZO film that annealed by rapid thermal annealing at 600 °C in N_2_ for 60 s. It indicated that the resistance of the ALD-AZO film was much smaller than that of the PAD-AZO film. However, the contact between ALD-AZO and p-GaN was non-ohmic. The ALD-AZO films were annealed by RTA in N_2_ (for not only 60 s, data not shown), the contacts between ALD-AZO and p-GaN were still non-ohmic, so the PAD-AZO layer was needed. The resistivity of PAD-AZO (30 nm) and two-step AZO (150 nm) were 2.221 × 10^−3^ Ω·cm and 2.175 × 10^−3^ Ω·cm. It was difficult for PAD method to grow thick AZO films with low resistance and the thickness of 30 nm may be a little thin for electrodes. So in this case, PAD-AZO was used to form ohmic contact, and ALD-AZO was added to reduce the sheet resistance. While there was a slight improvement in resistivity, the sheet resistance had been greatly reduced to 145 Ω/sq. when ALD method was introduced. An important parameter of ohmic contact is related to the specific contact resistance (R_c_). Figure 3b shows the raw data of the specific contact of the PAD-AZO (without ALD-AZO) and two-step AZO (with ALD-AZO) to extract specific contact resistance, the inner graph shows the structure of CTLM, the inner dot radius was 100 um, and the space between the inner and the outer radius was varied from 5 to 30 um. From the data the specific contact resistance can be calculated, the equations are R_m_ ≈ R_sh_[ln((r + s)/r)]/2π +L_T_R_sh_ln[(2r + s)/r(r + s)]/2π and R_c_ ≈ R_sh_·L_T_^2^, where R_m_ stands for the resistance between two electrodes, r stands for inner radius, L_T_ stands for transfer length, in the Fig. 3b, c = (r/s)*ln((r + s)/r), s stands for the spacing between inner and outer contact. The lowest specific contact resistance of the PAD-AZO films was about 1.08 × 10^−1^ Ω·cm^2^, and the lowest specific contact resistance of the two-step deposited AZO film was about 1.47 × 10^−2^ Ω·cm^2^. In our opinion, the reduction of the specific contact resistance was attributed to that the resistivity of ALD-AZO films was lower than that of PAD-AZO films, which may be caused by the dopant of hydrogen atoms [8, 29]. At the same time, the resistance between indium electrode and ALD-AZO was smaller than that between indium electrode and PAD-AZO. The resistance measured by I-V test (contained contact resistance) was larger than that measured by van der pauw geometry, the difference between these two resistances in PAD-AZO (1200 Ω) was larger than the difference in ALD-AZO (300 Ω).

**Fig. 4:**
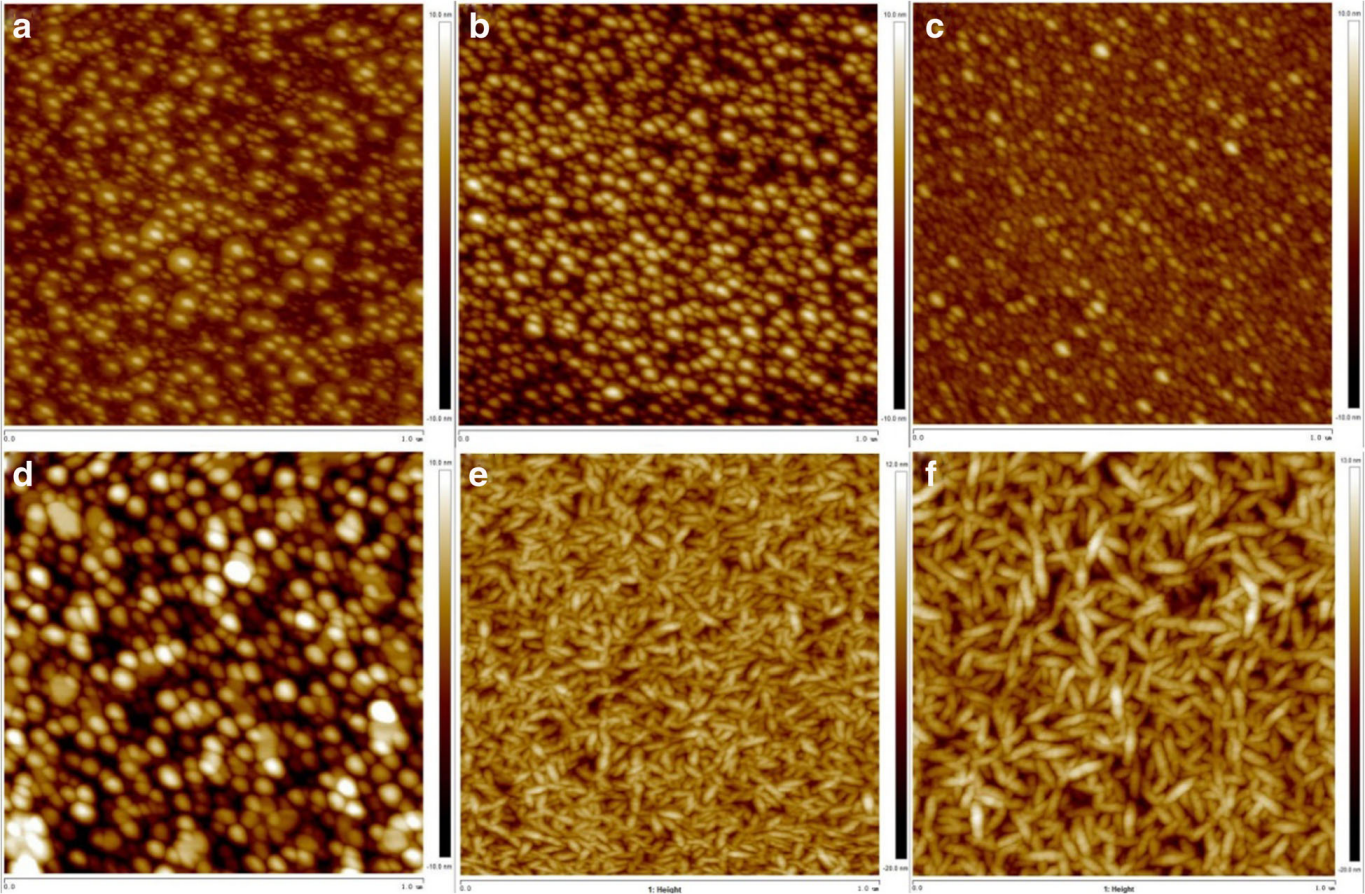
**a**, **b**, **c**, **d** Surface morphologies of PAD-AZO films (1 μm × 1 μm) at different growth temperatures of 500, 600, 700 and 800 °C, respectively. **e** the surface morphologies of ALD-AZO film that directly on p-GaN. **f** the surface topography of two-step deposited AZO film

Figure 4 shows the surface topography of the PAD-AZO films at different growth temperatures (a) 500, (b) 600, (c) 700 and (d) 800 °C, respectively. It can be observed that AZO started to form on the substrate at 500 °C. The AZO grains were uniform and compact when the growth temperature was 600 °C, with an average grain size of about 70 nm. However at 700 °C, some of the grains grew up at the expense of the others. When the growth temperature reached 800 °C, the grains became larger. Considering the effect of growth temperature and the resistivity, 600 °C was chosen as the proper growth temperature. Figure 4e shows the surface topography of the ALD-AZO film directly on p-GaN, and Fig. 4f shows the surface topography of the two-step deposited AZO film. It can be concluded from (e) and (f) that although the grain size changed, the structure was still mosaic. This change may be attributed to the insertion of the PAD-AZO interlayer to reduce the lattice mismatch.

**Fig. 5:**
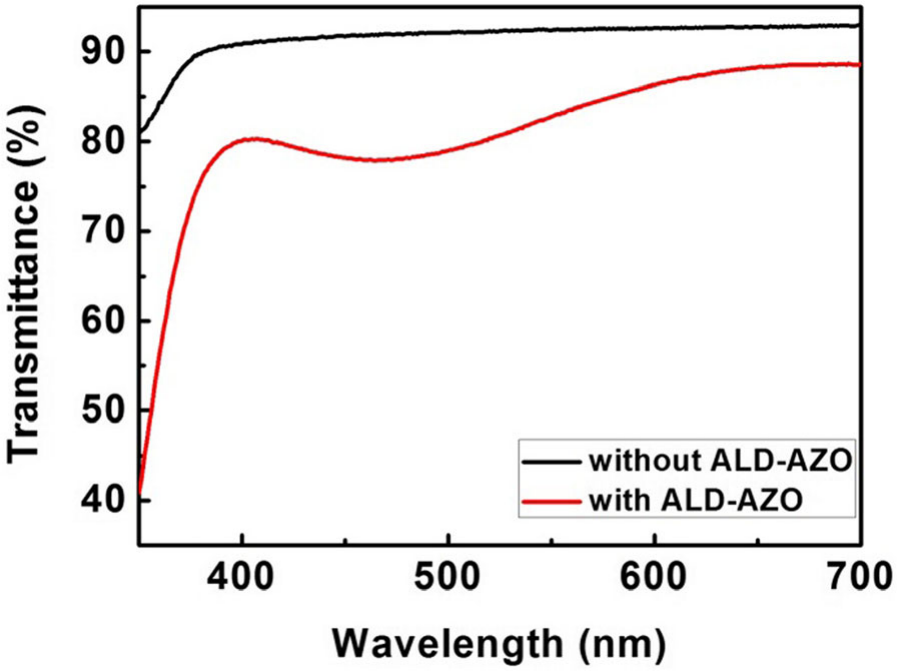
The transmittances of the PAD-AZO films and the two-step AZO films

Figure 5 shows the transmittances of the AZO films with and without ALD-AZO layer. The growth conditions on quartz were kept the same as those on p-GaN. The transmittance spectra for the PAD-AZO films were nearly the same for all samples with the value over 90% in the wavelength range of 400–700 nm, corresponding to the visible light. Although the transmittance reduced to about 80% when the ALD-AZO was deposited on the PAD-AZO films, the transmittances were still much higher than that of the oxidized Ni/Au films (55–70% in the visible range) [30] and nearly the same with the transmittance of ITO films [31].

## Conclusions

In this study we have successfully prepared AZO thin films on p-GaN by a combination of PAD and ALD method. The AZO thin films were (002) oriented, and highly transparent (about 80%) in the wavelength range of 400–700 nm. The optimal resistivity was 2.175 × 10^−3^ Ω·cm and the lowest specific contact resistance of the two-step deposited AZO film was about 1.47 × 10^−2^ Ω·cm^2^. Our results show that the two-step method can be used to prepare transparent and conducting AZO electrodes for industrial application.
